# Supplementary material for: Automated retinal disease classification using deep learning and AlexNet with statistical models analysis
Source: PLoS One. 2026 Jan 6;21(1):e0338415. doi: 10.1371/journal.pone.0338415 (PMC12774357; doi:10.1371/journal.pone.0338415)
Supplement: Attachment — Submitted filename: point bt point reviwer .docx [file pone.0338415.s001.docx]

**Reply to Reviewers Comments**

**Reviewer 1**

**Comment 1:** The abstract should describe the motivation for choosing AlexNet and EfficientNet, emphasizing their architectural differences and rationale for comparison.

**Response:** We thank the reviewer for this valuable suggestion. The abstract now explains the motivation behind selecting AlexNet and EfficientNet, highlighting their architectural differences, computational efficiency, and comparative purpose.

**Comment 2:** Include the key statistical validation (e.g., Wilcoxon signed-rank test) results concisely to demonstrate analytical rigor.

**Response:** We thank the reviewer for the insightful comment. The abstract and results sections now include the Wilcoxon signed-rank test results and significance values to emphasize statistical validity.

**Comment 3:** Provide clearer motivation linking clinical significance (e.g., blindness prevention statistics) to computational necessity.

**Response:** We thank the reviewer for this important recommendation. A new paragraph in the introduction now links blindness prevention and clinical relevance to the computational necessity of automated retinal diagnosis.

**Comment 4:** Emphasize the research gap more explicitly and justify AlexNet’s inclusion despite its older architecture.

**Response:** We thank the reviewer for highlighting this point. The “Research Gap and Main Contribution” section has been rewritten to clarify the limitations of prior works and justify AlexNet’s inclusion as a lightweight, efficient benchmark.

**Comment 5:** Include more recent epidemiological data (2024 WHO Vision Report).

**Response:** We thank the reviewer for this suggestion. The introduction has been updated with statistics from the 2024 WHO Vision Report to enhance credibility and context.

**Comment 6:** Add a critical comparative discussion rather than descriptive summaries in Table 1.

**Response:** We thank the reviewer for this constructive comment. Table 1 now includes analytical comparisons of prior methods, highlighting methodological differences, dataset diversity, and observed limitations.

**Comment 7:** Integrate transformer-based or hybrid vision architectures (e.g., Vision Transformers, ConvNeXt) to align with 2023–2025 research trends.

**Response:** We thank the reviewer for this valuable insight. Transformer-based and hybrid vision studies have been incorporated into the Related Works section for completeness. Their experimental implementation will be part of future work.

**Comment 8:** Clarify citation redundancy (e.g., ref [9] and [16] appear repeated).

**Response:** We thank the reviewer for pointing this out. Duplicate citations have been carefully reviewed and corrected.

**Comment 9:** Clarify hardware configuration (GPU type, memory, training time) and optimizer parameters (learning rate, batch size, epochs).

**Response:** We thank the reviewer for the suggestion. The Materials and Methods section now clearly specifies GPU type, memory, optimizer, learning rate, batch size, and number of epochs.

**Comment 10:** Include confusion matrices for all models.

**Response:** We thank the reviewer for this helpful comment. Confusion matrices for alexnet model have been added to the Results section for a clearer performance evaluation.

**Comment 11:** Report confidence intervals or statistical significance across cross-validation folds.

**Response:** We thank the reviewer for the insightful recommendation. Confidence intervals have been included where applicable. Full k-fold cross-validation will be conducted in future work to enhance statistical reliability.

**Comment 12:** Incorporate ROC and AUC metrics for a more comprehensive diagnostic evaluation.

**Response:** We thank the reviewer for this valuable point. We will add this in future work.

**Reviewer 2**

**Comment 1:** Add k-fold cross-validation (5- or 10-fold) to verify generalizability and minimize dataset bias.

**Response:** We thank the reviewer for this important suggestion. K-fold cross-validation will be implemented in future work to improve model generalization and reduce dataset bias.

**Comment 2:** Report training time, number of parameters, and FLOPs (Floating Point Operations) for each model.

**Response: W**e thank the reviewer for this recommendation. Training time and parameter countss have been added in the Results section to demonstrate the balance between efficiency and performance.

**Comment 3:** Apply visualization methods such as Grad-CAM, LIME, or SHAP to show how models localize disease regions.

**Response:** We thank the reviewer for this insightful comment. SHAP visualizations have been added to demonstrate disease localization.

**Comment 4:** Include error analysis for misclassified samples.

**Response:** We thank the reviewer for the helpful suggestion. An error analysis section has been added.

**Comment 5:** Deepen the Related Works section by including recent (2023–2025) literature on transformer-based retinal models, attention mechanisms, and multimodal learning.

**Response:** We thank the reviewer for this valuable recommendation. The Related Works section has been expanded to include recent studies from 2023–2025, focusing on transformer-based and multimodal approaches**.**

**Comment 6:** Discuss challenges such as model interpretability, hardware constraints in clinics, and regulatory compliance for medical AI systems.

**Response:** We thank the reviewer for the important observation. The Discussion section now includes a paragraph addressing interpretability challenges, clinical hardware limitations, and regulatory aspects related to medical AI deployment.
